# Reliability of electric field simulations in different age groups: impact of data quality metrics

**DOI:** 10.1186/s40708-026-00293-2

**Published:** 2026-02-22

**Authors:** Dayana Hayek, Axel Thielscher, Ulrike Grittner, Agnes Flöel, Daria Antonenko

**Affiliations:** 1 Department of Neurology, University Medicine Greifswald, Ferdinand-Sauerbruch-Straße, 17475 Greifswald, Germany; 2 Department of Health Technology, Technical University of Denmark, Kongens Lyngby, Denmark; 3 Department of Radiology and Nuclear Medicine, Danish Research Centre for Magnetic Resonance, Copenhagen University Hospital Amager and Hvidovre, Hvidovre, Denmark; 4 Charité – Universitätsmedizin Berlin, Humboldt-Universität Zu Berlin, Berlin Institute of Health, Institute of Biometry and Clinical Epidemiology, 10117 Berlin, Germany; 5 German Centre for Neurodegenerative Diseases (DZNE) Standort Greifswald, Greifswald, Germany

**Keywords:** Electric field modeling, MRI quality, Transcranial direct current stimulation, Aging, Finite element modeling

## Abstract

Electric field simulations based on individualized Magnetic Resonance Imaging (MRI)-derived head models are increasingly used to optimize non-invasive brain stimulation (NIBS) protocols, enabling individualized dose adjustments to achieve a desired current flow at cortical targets. However, the quality of structural MRI data—affected by motion artefacts, tissue contrast, and scanner-related noise—may influence the accuracy of these simulations, an issue possibly exacerbated in older adults who often exhibit lower image quality. If reduced image quality compromises electric field estimation, it could limit the feasibility of individualized dosing in aging populations. In this study, we examined whether standardized image quality metrics—entropy focus criterion (EFC), signal-to-noise ratio (SNR), contrast-to-noise ratio (CNR), spatial resolution (FWHM), and intensity non-uniformity (INU)—systematically relate to the magnitude of simulated electric fields in young and older adults. We analysed MRI and simulation data of a focal C3 montage from 106 healthy adults, 47 young adults (mean age: 24.8, age range: 20–35 years) and 59 older adults (mean age: 69.5 years, age range: 60–79) using SimNIBS for computational modelling of the electric fields and MRIQC for image quality assessment. Structural equation modelling was used to quantify direct and indirect effects of age group on electric field magnitude, with image quality as a mediating factor. Head volume was included in extended models to control for anatomical variation. Our findings demonstrate that MR image quality is associated with simulated electric field magnitude, with higher EFC, lower SNR, lower CNR, and higher INU associated with reduced field estimates, and older adults showing generally lower simulated electric fields compared to younger adults. While image quality accounted for some of the age-related differences in electric field strength, group differences remained even after controlling for EFC, SNR and head volume. Critically, electric field simulations remained sufficiently reliable despite lower image quality in older adults, supporting their use for individualized dose adjustment across the lifespan.

**Supplementary Information:**

The online version contains supplementary material available at 10.1186/s40708-026-00293-2.

## Introduction

Non-invasive brain stimulation techniques such as transcranial magnetic stimulation and transcranial direct current stimulation (tDCS) are established approaches to modulate brain activity, for enhancing cognitive functions, and promoting neuroplasticity [1, 2]. A critical step in optimizing these interventions involves accurate simulation of the electric fields generated within the brain, which determines the effective cortical dose delivered by stimulation [3–5]. Such simulations are essential for individualized dosing strategies, both in research and in therapeutic applications, because anatomical and physiological variability—including age-related changes—can substantially alter the effective cortical dose at a given current intensity [6, 7]. Finite element modeling (FEM) is the standard computational approach used for this purpose, relying on structural head and brain images—typically from magnetic resonance imaging (MRI)—to create individualized head models that capture each participant’s unique anatomical features. These models enable estimation of the spatial distribution and magnitude of individual electric fields, providing an essential basis for dose–response research and for developing adaptive stimulation protocols [8, 9]. Previous studies using FEM have reported differences related to age and neurodegenerative disease, showing that resultant estimates of individual cortical dose of the applied brain stimulation protocol emerged as potential biomarker for response to the intervention [10, 11].

The accuracy of these simulations depends critically on image segmentation quality, which is influenced by MRI acquisition parameters and subject-related factors such as head motion [12, 13]. When image quality of structural MRI data is affected by motion-related artefacts, this can compromise tissue segmentation and morphometric accuracy [14–16]. For example, head motion during MRI acquisition has been linked to systematic underestimation of cortical thickness and grey matter volume, confounding associations with age or clinical variables [17]. This was also observed in other MRI sequences, for example, Geerligs et al. demonstrated that functional connectivity estimates are influenced by non-neural confounds such as head motion, leading to apparent group differences that may be mistaken for neural effects [15]. These findings illustrate how image quality can bias derived imaging metrics and raise the question of whether similar biases occur in electric field simulations.

A prominent bias in aging research is that older adults often exhibit factors such as greater head motion and age-related anatomical or physiological changes, leading to systematic differences in MRI quality [15, 18, 19]. This is particularly relevant for FEM-based simulations because aging studies have reported lower E-field magnitudes for the same applied current [20, 21]. Clarifying whether these reductions reflect true anatomical changes or are partly driven by MRI quality differences is essential. Among widely used quality metrics, entropy focus criterion (EFC), signal-to-noise ratio (SNR), contrast-to-noise ratio (CNR), spatial resolution (corresponding to full-width-at-half-maximum, FWHM), and intensity non-uniformity (INU) capture distinct aspects of image quality. EFC, based on entropy, is strongly related to motion artifacts, which are more prevalent in older adults [22–24]. SNR reflects signal clarity relative to noise; while strongly influenced by acquisition and hardware factors, age-related changes such as increased physiological noise and brain atrophy can also reduce SNR [15, 25, 26]. In contrast, CNR indexes gray–white matter contrast and often declines with age due to anatomical changes such as cortical thinning and reduced tissue differentiation [19, 27–29]. FWHM reflects the FWHM of the spatial distribution of the image intensity values [30], and INU represents the median of the bias field [31], which may as well be different between age groups. Together, these metrics provide an aging-relevant framework for assessing structural image quality. This raises the question of whether differences in MRI quality systematically bias electric field simulations, particularly in aging populations where such differences are pronounced.

The present study addresses this gap by systematically examining (i) whether MRI quality metrics are associated with simulated E-field magnitudes, (ii) whether group differences in E-fields are explained by image quality, and (iii) whether simulations remain reliable despite poorer image quality in older adults. Our overarching goal was to assess the reliability of E-field modeling across the adult lifespan and its suitability for guiding personalized brain stimulation protocols. We used established quality control metrics—EFC, SNR, CNR, FWHM, and INU—to assess structural MRI quality, and applied individualized FEM using SimNIBS [32] to model electric fields. Using structural equation modeling, we examined both direct and indirect effects of age group on electric field estimates, with image quality as a mediating factor. In extended models, we also included head volume as a covariate, given its known associations with both motion artifacts and electric field distribution [17, 20]. By systematically quantifying these effects, our findings contribute to a more nuanced understanding of how imaging quality, head volume, and age-related structural differences impact the reliability of electric field simulations. This has important implications for the development of accurate, personalized NIBS interventions, particularly in aging populations.

## Methods

### Participants

This study included 106 participants, divided into two groups based on age: 47 young adults (age range: 20–35 years, mean age ± SD: 24.8 ± 3.5 years, 59.6% female) and 59 older adults (age range: 60–79 years, mean age ± SD: 69.5 ± 4.2 years, 61% female). All participants underwent a screening process to ensure they had no neurological or psychiatric conditions that would contraindicate their participation in the study, particularly regarding magnetic resonance imaging (MRI). All participants provided written informed consent after receiving a full explanation of the study procedures, in accordance with the Declaration of Helsinki.

### Magnetic resonance imaging (MRI) acquisition

Structural MRI data were acquired using two 3-Tesla Siemens scanners (MAGNETOM Verio and MAGNETOM Skyra), each with a 32-channel head coil. T1-weighted images were collected using a 3D MPRAGE sequence with TR = 1690–2300 ms, TE = 2.52–2.96 ms, TI = 900 ms, voxel size = 1.0 × 1.0 × 1.0 mm^3^, flip angle = 9°, and field of view = 250–256 × 240–250 mm^2^. Selective water excitation was used for fat suppression. T2-weighted images were acquired using a 3D TSE sequence with TR = 12,770 ms, TE = 85.0–86.0 ms, flip angle = 111°, voxel size = 1.0 × 1.0 × 1.0–1.1 mm^3^, and field of view = 256 × 240 mm^2^. These scans provided the anatomical data to create individualized head models for electric field simulations.

### Image quality metrics

The image quality of the MRI scans was assessed using the MRI Quality Control (MRIQC) tool [23], a widely used software designed to evaluate the quality of MR images. Specifically, the tool provided key metrics such as Entropy-focus Criterion (EFC), Signal-to-Noise Ratio (SNR), Contrast-to-Noise Ratio (CNR), spatial resolution (corresponding to full-width-at-half-maximum, FWHM), and intensity non-uniformity (INU).

MRIQC was utilized to extract image quality metrics for each participant. EFC, a whole-image metric based on the Shannon entropy of voxel intensities, was calculated using the entire image volume and a frame mask identifying empty voxels after rotation [22]. SNR was calculated separately within gray matter (GM), white matter (WM), and cerebrospinal fluid (CSF) masks, as the mean signal intensity divided by its standard deviation in each tissue compartment [25]. CNR, defined as the contrast between gray and white matter signal intensities relative to background noise, was calculated using the white matter, gray matter, and air masks [27]. Please note that the gray-to-white matter contrast is known to be age-dependent, making CNR unsuited as unbiased estimate of image quality across the age range. The FWHM of the spatial distribution of the image intensity was extracted in units of voxels. Lower values are better, higher values indicate a blurrier image [30]. Median of the INU field (bias field) was extracted by the N4ITK algorithm [31]. Values closer to 1 are better, values further from zero indicate greater RF field inhomogeneity. These metrics allowed for an in-depth comparison of image quality across the two age groups and provided the foundation for further analyses of their potential impact on the subsequent electric field modeling.

### Electric field estimation using finite element modeling (FEM)

**Fig. 1:**
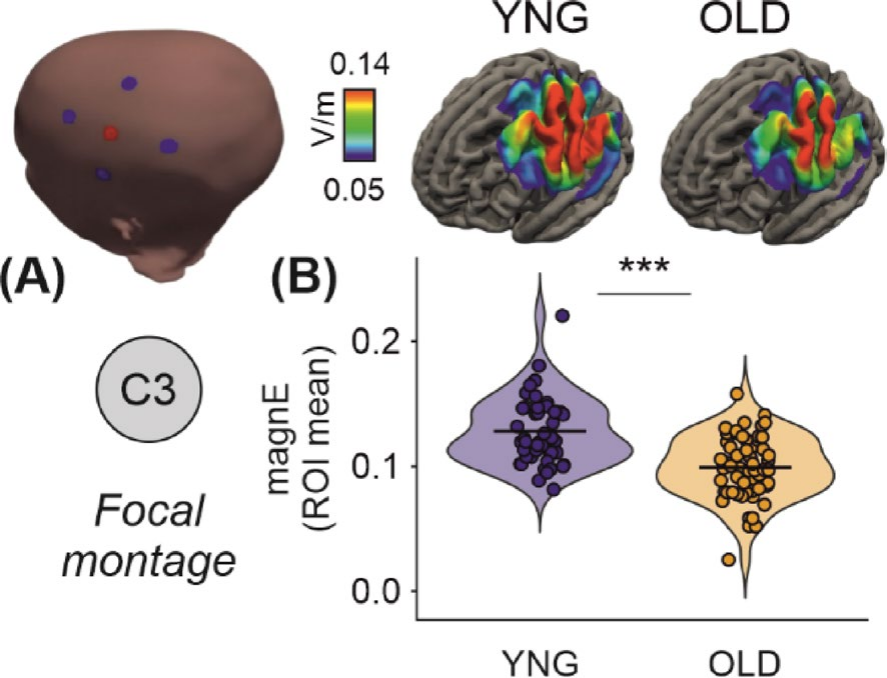
Differential electric field distribution and magnitude in young (n = 47) and old (n = 59) adults using focal tDCS montage. **A** Illustration of the focal transcranial direct current stimulation (tDCS) montage targeting the C3 region (left) and simulated group-average electric field distributions in young and old adult brains (middle and right, respectively). The heatmaps show electric field strength, with the scale ranging from 0.050 to 0.140 V/m. **B** Violin plot comparing the mean electric field magnitudes between young and old adults, revealing significantly higher field magnitudes in young adults (***p < 0.001). Each dot represents an individual simulation. Horizontal lines indicate the group means

Finite element modelling was employed to simulate the electric fields induced by transcranial electrical stimulation (tES) in each participant. Using the SimNIBS version 4.0.1 toolbox [32], individual head models in form of tetrahedral meshes were created using the CHARM pipeline utilizing the T1- and T2-weighted MRI scans of the participants, resulting in tetrahedral finite element meshes containing 4.11 ± 0.28 million elements for younger adults (n = 47) and 3.91 ± 0.32 million elements for older adults (n = 59). These meshes included detailed representations of the scalp, skull, spongy bone, cerebrospinal fluid, gray matter, white matter, eyes and large blood vessels). Tissue conductivities were assigned according to SimNIBS defaults: white matter = 0.126 S/m, gray matter = 0.275 S/m, cerebrospinal fluid = 1.654 S/m, compact bone = 0.008 S/m, spongy bone = 0.025 S/m, scalp = 0.465 S/m, eyes = 0.5 S/m, and blood vessels = 0.6 S/m [6, 33]. Isotropic conductivities were applied throughout all tissue compartments, as diffusion tensor imaging data for anisotropic conductivity modeling were not available. For the focal montage, a 4 × 1 ring configuration was implemented with one central 10 mm diameter anode at C3 surrounded by four 10 mm diameter return cathodes at C1, C5, FC3, and CP3, following the 10–10 EEG coordinate system, all with 5 mm saline gel layers. Electrodes were modeled using the complete electrode model with equipotential boundary conditions at electrode surfaces. A total current of 2 mA was applied, with 2 mA injected at the central electrode and − 0.5 mA returned through each of the four surrounding electrodes. This montage, commonly used to target the left primary motor cortex, was used to estimate electric field distributions and their variability across participants and age groups [34, 35]. In addition to the C3 focal montage, five further configurations were simulated: focal montages centered at F3 and P3, and conventional pad montages with anodes placed at F3, P3, or C3 and a common cathode at AF4. For the focal montages, the return electrodes were positioned at AF3, FC3, F1, and F5 (F3 center), or CP3, PO3, P1, and P5 (P3 center), using the same 10 mm diameter circular electrodes and the same total current intensity (2 mA, distributed as + 2 mA at the center, − 0.5 mA per return electrode). For the conventional montages, two 50 × 50 mm rectangular electrodes were modeled with a 5 mm saline gel layer (conductivity = 1.0 S/m) with their center aligned to the designated EEG position (F3, P3, or C3 for the anode; AF4 for the cathode). These electrodes were oriented tangentially to the scalp surface and followed the head curvature. A bipolar configuration was used, with 2 mA injected at the anode and − 2 mA at the cathode. All montages were processed using the same modeling pipeline. The resulting electric field distributions for younger and older adults across all configurations are shown in Supplementary Figs. 1–5. Electric field magnitudes were extracted from regions-of-interest (ROI) below the electrodes (averaging the magnitudes in the target regions: left primary motor, left dorsolateral prefrontal, and left posterior parietal region). These simulations generated estimates of the induced electric field magnitudes, which were then analysed for their variability across participants and age groups (Fig. 1A). To ensure robustness of the results and path models, the 95th and 99th (corresponding to the peak values) percentiles were extracted and compared in complementary analyses (Supplementary Figs. 6–7).

Head tissue volumes (combining WM, GM, CSF, compact bone, spongy bone, skin, eye and blodd vessels volumes) were derived from the individual tetrahedral head meshes. Each mesh was reoriented using an anatomical coordinate system defined by fiducial landmarks: nasion (Nz), inion (Iz), left preauricular point (LPA), and right preauricular point (RPA). A custom cut-off plane was applied at 20 mm superior to this reference plane (corresponding to z = − 20 mm in the transformed coordinate system). Only mesh elements located above this plane were retained for volume computation. This restriction ensured consistent comparison across individuals by focusing on the cranial region. Region-specific tissue volumes were then extracted from the resulting surface-cut meshes using SimNIBS-compatible processing tools.

### Statistical analysis

To investigate the impact of MRI quality on the electric field magnitudes, Structural Equation Modeling (SEM) was used. SEM was conducted using the Lavaan package in R [36, 37], which allows for the modelling of complex relationships between variables. In this case, we assessed the relationship between MRI quality metrics (CNR, SNR, EFC, FWHM, and INU) in both T1- and T2-weighted images and electric field magnitudes while accounting for the differences between young and older adults. Additionally, to examine potential confounding effects of anatomical differences, head volume was included as an additional covariate in extended SEM models. The SEM path models enabled us to estimate both direct and indirect effects of age group on the electric field magnitudes, considering the mediating effects of image quality metrics. Models were tested in two steps: (1) single-mediator models including each MRI quality metric individually, and (2) extended models incorporating head volume along with the quality metrics to adjust for individual differences in head volume. Standardized coefficients (ß) were calculated to provide a direct and comparable measure of effect sizes. This approach enables the quantification of electric field strengths between the two age groups, independent of the effects of image quality. To ensure robustness, we further conducted stratified analyses of T1 image quality metrics (EFC, SNR, CNR, FWHM, and INU) using a median-split approach within each target (C3, F3, P3) and montage (focal, conventional). Age-group effects on electric field magnitude (ROI mean) were estimated separately within each quality stratum, and formal Group × Quality interaction tests were conducted.

### Ethical considerations

The research received approval from the Ethics Committee of University Medicine Greifswald (BB 004/18, BB 002/21, BB 021/19). All procedures were conducted in accordance with the Helsinki Declaration, and written informed consent was obtained from all participants prior to their involvement in the study. The consent process ensured that participants understood the nature of the study, the procedures involved, and their right to withdraw at any time without penalty.

## Results

### Group differences in image quality metrics

**Fig. 2:**
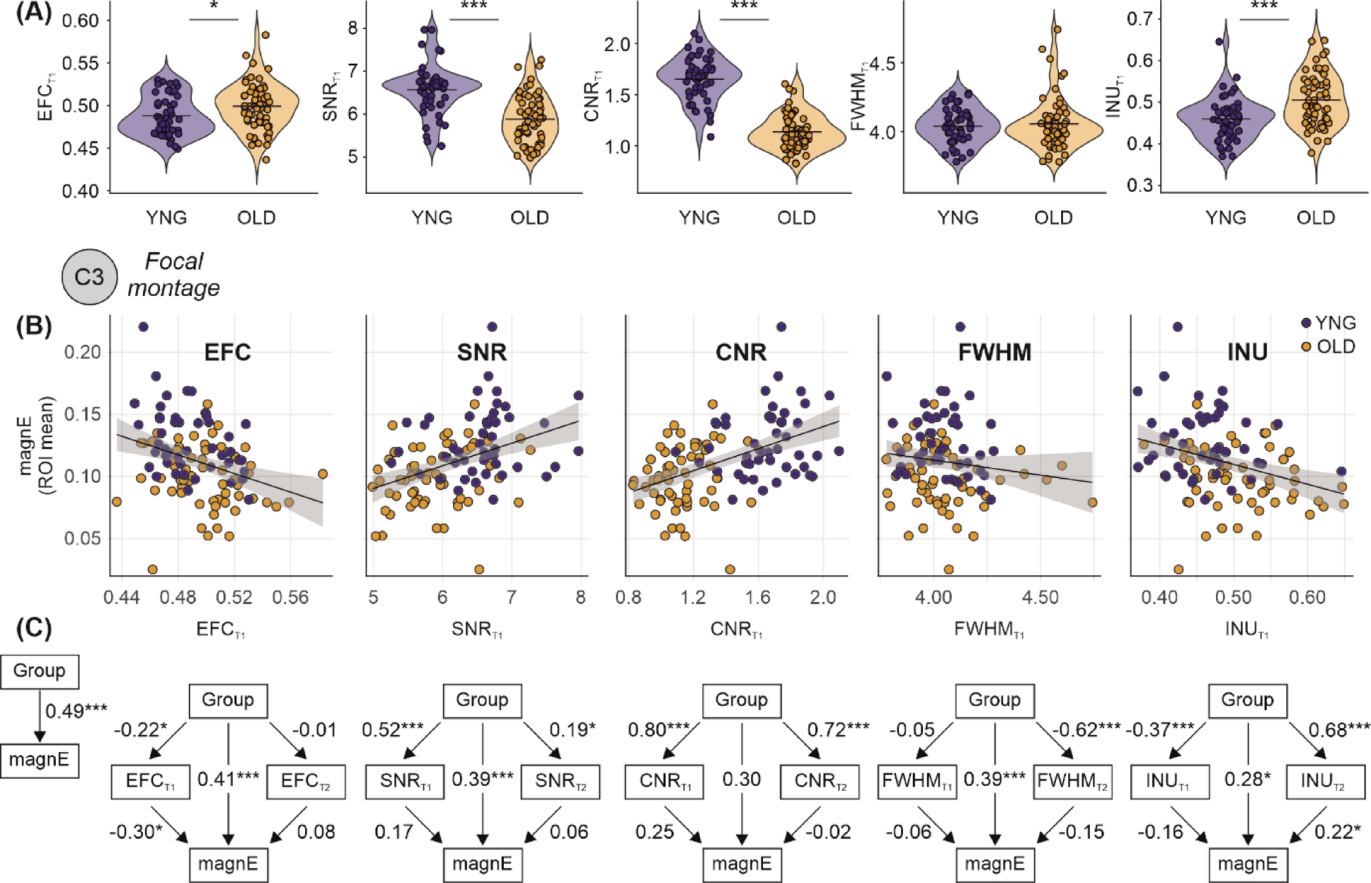
Age-related differences in brain tissue properties and their mediation of electric field magnitude. **A** Violin plots comparing image quality metrics in T1-weighted image—Entropy Focus Criterion (EFC), Signal-to-Noise Ratio (SNR), and Contrast-to-Noise Ratio (CNR)—between young (n = 47) and old (n = 59) adults. Older adults showed significantly lower SNR and CNR and higher EFC. **B** Scatter plots showing associations between electric field magnitude and EFC, SNR, CNR, FWHM, and INU in T1-weighted images across age groups. Regression lines show relationships indicating that tissue properties are associated to electric field distribution. **C** Structural Equation Models (SEMs) illustrating the direct and indirect effects of age group on electric field magnitude mediated by image quality metrics in both T1- and T2-weighted images (EFC, SNR, CNR, FWHM, and INU). Each model shows that age groups differed in the respective image quality metric, which in turn partially mediates electric field estimates. Critically, the direct association between age group and electric field magnitude remains after accounting for EFC, SNR, FWHM, and INU. For CNR, adjustment attenuated the group effect and mediation was not significant, likely reflecting its strong dependence on age-related tissue contrast. This indicates that age-related differences in electric field magnitude are only partially attributable to image quality and likely reflect additional biological or anatomical differences between groups. Standardized path coefficients are shown with *p*-values: *p < 0.05, **p < 0.01, ***p < 0.001

We examined the unadjusted effects of group on individual MRI quality metrics using structural equation modelling (SEM). Young adults showed higher electric field magnitude (β = 0.486, *p* < 0.001, 95% CI [0.350, 0.623]) compared to older adults (Fig. 1B, Table 1). In addition, standardized regression analyses revealed group effects on all quality T1 measures except spatial resolution (Fig. 2A). Young adults showed lower EFC (β = − 0.216, *p* = 0.018, 95% CI [− 0.395, − 0.036]), higher CNR (β = 0.801, *p* < 0.001, 95% CI [0.744, 0.857]), higher SNR (β = 0.517, *p* < 0.001, 95% CI [0.387, 0.647]), and higher INU (β = − 0.369, *p* < 0.001, 95% CI [− 0.533, − 0.204]) compared to older adults, indicating higher image quality.

**Table 1:** Standardized path coefficients for group effects on e-field magnitude through interaction with image quality metrics (n = 106)

Model	Estimate (Std.)	Z-value	*P*-value	95% CI lower	95% CI upper
*Entropy focus criterion (EFC)*					
Effect of Group on E-Field Magnitude	0.486	6.977	< 0.001	0.350	0.623
Effect of Group on T1 EFC	− 0.216	− 2.356	0.018	− 0.395	− 0.036
Effect of Group on T2 EFC	− 0.009	− 0.091	0.927	− 0.199	0.182
Effect of T1 EFC on E-field Magnitude	− 0.295	− 3.715	< 0.001	− 0.451	− 0.140
Effect of T2 EFC on E-field Magnitude	0.082	1.024	0.306	− 0.075	0.239
Effect of Group on E-field Magnitude (adjusted for T1 & T2 EFC)	0.414	5.677	< 0.001	0.271	0.557
*Signal-to-noise ratio (SNR)*					
Effect of Group on E-Field Magnitude	0.486	6.977	< 0.001	0.350	0.623
Effect of Group on T1 SNR	0.517	7.796	< 0.001	0.387	0.647
Effect of Group on T2 SNR	0.188	2.021	0.043	0.006	0.370
Effect of T1 SNR on E-field Magnitude	0.167	1.729	0.084	− 0.022	0.356
Effect of T2 SNR on E-field Magnitude	0.056	0.665	0.506	− 0.110	0.223
Effect of Group on E-field Magnitude (adjusted for T1 & T2 SNR)	0.391	4.297	< 0.001	0.213	0.570
*Contrast-to-noise ratio (CNR)*					
Effect of Group on E-Field Magnitude	0.486	6.977	< 0.001	0.350	0.623
Effect of Group on T1 CNR	0.801	27.878	< 0.001	0.744	0.857
Effect of Group on T2 CNR	0.720	17.924	< 0.001	0.642	0.799
Effect of T1 CNR on E-field Magnitude	0.248	1.801	0.072	− 0.022	0.519
Effect of T2 CNR on E-field Magnitude	− 0.019	− 0.155	0.877	− 0.255	0.218
Effect of Group on E-field Magnitude (adjusted for T1 & T2 CNR)	0.300	1.854	0.064	− 0.017	0.617
*Full width at half maximum (FWHM)*					
Effect of Group on E-Field Magnitude	0.486	6.977	< 0.001	0.350	0.623
Effect of Group on T1 FWHM	− 0.046	− 0.471	0.637	− 0.236	0.144
Effect of Group on T2 FWHM	− 0.620	− 11.543	< 0.001	− 0.725	− 0.515
Effect of T1 FWHM on E-field Magnitude	− 0.060	− 0.720	0.472	− 0.225	0.104
Effect of T2 FWHM on E-field Magnitude	− 0.148	− 1.393	0.164	− 0.356	0.060
Effect of Group on E-field Magnitude (adjusted for T1 & T2 FWHM)	0.393	3.940	< 0.001	0.198	0.589
*Intensity non-uniformity (INU)*					
Effect of Group on E-Field Magnitude	0.486	6.977	< 0.001	0.350	0.623
Effect of Group on T1 INU	− 0.368	− 4.535	< 0.001	− 0.527	− 0.209
Effect of Group on T2 INU	0.682	14.991	< 0.001	0.593	0.771
Effect of T1 INU on E-field Magnitude	− 0.157	− 1.791	0.073	− 0.329	0.015
Effect of T2 INU on E-field Magnitude	0.221	1.993	0.046	0.004	0.439
Effect of Group on E-field Magnitude (adjusted for T1 & T2 INU)	0.279	2.450	0.014	0.056	0.502

All coefficients are standardized. Model 1 shows the unadjusted effect of Group (young vs. old) on E-field magnitude. Model 2 shows effects adjusted for image quality metrics at T1 and T2. Group coded as 1 = young, 0 = old. C3 = central electrode position; Focal montage = 4 × 1 ring configuration

### Path models: indirect effects via image quality metrics

To further explore the mechanisms through which group differences in electric field magnitude may be influenced by image quality, we tested path models incorporating each image quality metric (EFC, SNR, CNR, FWHM, and INU) from both T1- and T2-weighted images as mediators. Our analysis revealed that age group differences in electric field magnitudes were partially mediated by differences in image quality. These differences remained statistically significant after accounting for EFC, SNR, FWHM, and INU. Specifically, older adults had higher T1-derived EFC (*β* = − 0.216, 95% CI [− 0.395, − 0.036], *p* = 0.018), and higher EFC was associated with lower electric field magnitude (β = − 0.295, 95% CI [− 0.451, − 0.140], *p* < 0.001), indicating that higher image blur in older individuals is associated to lower estimated field strength. In contrast, T2-derived EFC showed no significant association with group (β = − 0.009, 95% CI [− 0.199, 0.182], *p* = 0.927) or electric field magnitude (β = 0.082, 95% CI [− 0.075, 0.239], *p* = 0.306), suggesting negligible influence from T2 image blur. Older adults also had lower T1-derived SNR (β = 0.517, 95% CI [0.387, 0.647], *p* < 0.001) and lower T2-derived SNR (β = 0.188, 95% CI [0.006, 0.370], *p* = 0.043). Higher T1 SNR was associated with higher electric field magnitude (β = 0.167, 95% CI [− 0.022, 0.356], *p* = 0.084), with no association for T2 SNR (β = 0.056, 95% CI [− 0.110, 0.223], *p* = 0.506), suggesting that lower image SNR—especially in T1—may inflate field estimates. Similarly, older adults had lower T1-derived CNR (β = 0.801, 95% CI [0.744, 0.857], *p* < 0.001) and lower T2-derived CNR (β = 0.720, 95% CI [0.642, 0.799], *p* < 0.001). While lower T1 CNR was associated with slightly higher electric field magnitude (β = 0.248, 95% CI [− 0.022, 0.519], *p* = 0.072), and T2 CNR showed no effect (β = − 0.019, 95% CI [− 0.255, 0.218], *p* = 0.877), suggesting that reduced tissue contrast may play a role. Importantly, after accounting for these indirect effects through image quality, the direct effect of age group on electric field magnitude remained strong and significant for EFC and SNR (EFC-adjusted: β = 0.414, 95% CI [0.271, 0.557], *p* < 0.001; SNR-adjusted: β = 0.391, 95% CI [0.213, 0.570], *p* < 0.001). For CNR, the direct effect was attenuated and no longer significant (β = 0.300, 95% CI [− 0.017, 0.617], *p* = 0.064), likely reflecting that CNR partly captures true age-related differences in tissue contrast rather than purely image quality. While T1- derived FWHM did not differ between groups (β = − 0.046, 95% CI [− 0.236, 0.144, *p* = 0.637), older adults had higher T2 FWHM (β = − 0.620, 95% CI [− 0.725, − 0.515], *p* < 0.001). INU for T1 (β = − 0.368, 95% CI [− 0.527, − 0.209], *p* < 0.001) and for T2 (β = 0.682, 95% CI [0.593, 0.771], *p* < 0.001) differed between age groups.

Across montages and field metrics, age-group differences in electric-field magnitudes were largely preserved after adjusting for image quality metrics. In most conditions, group effects remained comparable in magnitude across quality strata (see Supplementary Material for further details and Supplementary Table S1). For corrections involving CNR, group differences were reduced in several conditions (Table 2). Smaller attenuations were also observed sporadically for resolution (FWHM) and intensity non-uniformity (INU). In contrast, corrections based on EFC and SNR showed highly consistent group effects across all simulations and montages.

**Table 2:** Standardized path model estimates for the effects of group, EFC, and head volume on E-field magnitude (n = 106)

Model	Estimate (Std.)	Z-value	*P*-value	95% CI lower	95% CI upper
Effect of Head Volume on T1 EFC	0.794	21.68	< 0.001	0.722	0.866
Effect of Head Volume on T2 EFC	0.679	12.961	< 0.001	0.577	0.782
Effect of T1 EFC on E-field Magnitude	− 0.163	− 1.149	0.251	− 0.441	0.115
Effect of T2 EFC on E-field Magnitude	0.099	0.899	0.368	− 0.117	0.314
Effect of Group on T1 EFC	− 0.18	− 1.144	0.253	− 0.488	0.128
Effect of Group on T2 EFC	− 0.189	− 3.365	0.001	− 0.299	− 0.079
Effect of Head Volume on E-field Magnitude	0.014	0.194	0.846	− 0.126	0.154
Effect of Group on E-field Magnitude (adjusted for T1 EFC, T2 EFC & Head Volume)	0.444	5.676	< 0.001	0.291	0.597

For some simulations, group differences were small overall and remained so across quality corrections. These minor montage-specific variations emerged for the parietal target where age group differences and the impact of quality metrics showed some inconsistency, potentially due to greater inter-individual variability in parietal cortical anatomy [38] and CSF volumes in this region. Thus, reduced sensitivity in this montage reflects simulation-specific factors rather than image quality per se. Taken together, these findings support the conclusion that electric-field modeling remains robust across image quality variations, while highlighting metric- and montage-specific nuances.

These findings highlight that while motion-related and noise-related quality differences (EFC, SNR), spatial resolution (FWHM) and intensity non-uniformity (INU), only partially explain group effects, reductions in gray–white matter contrast with aging (captured by CNR) overlap with anatomical factors influencing E-field distribution.

### Path models: mediating roles of image quality and head volume

**Fig. 3:**
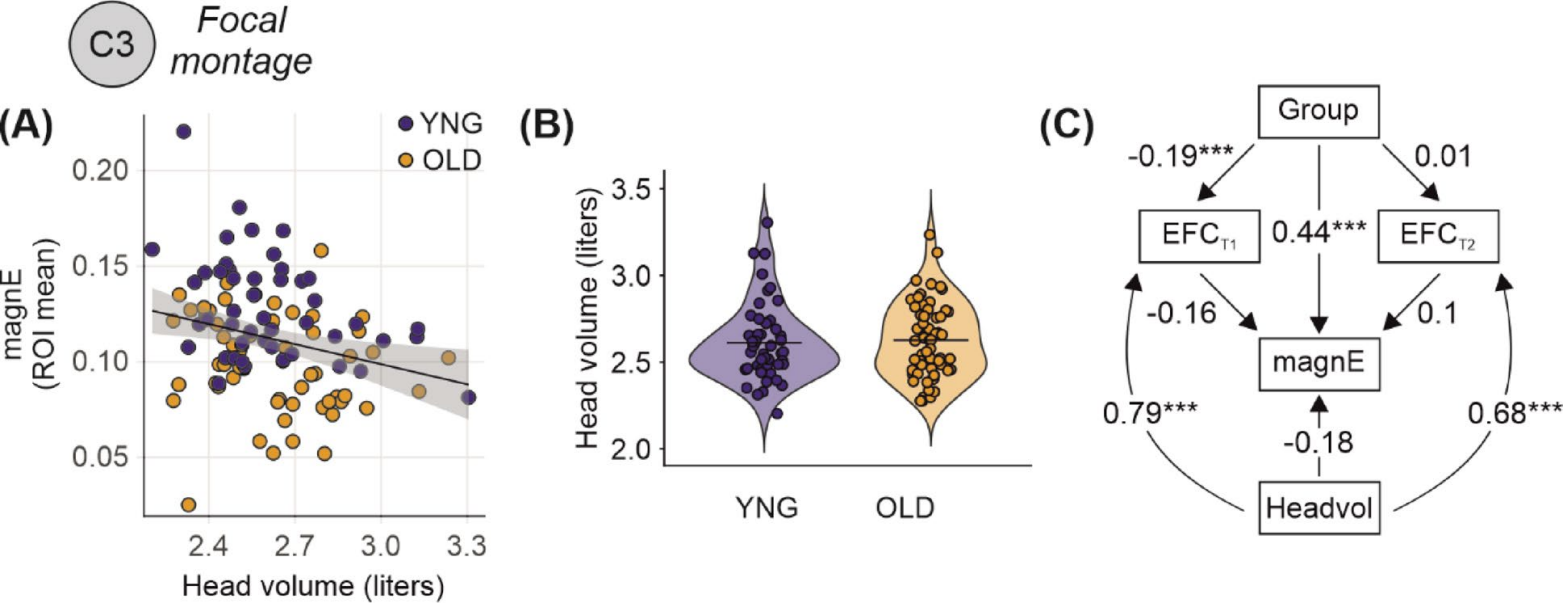
Head volume partially explains age differences in image quality but not electric field magnitude. **A** Scatter plot showing a negative association between head volume and electric field magnitude across participants, suggesting that individuals with larger head volumes tend to exhibit lower electric field strength. **B** Violin plots comparing head volume between young (n = 47) and old (n = 59) adults, indicating no group difference in head volume. **C** Structural Equation Model (SEM) evaluating the relationships among group, head volume, T1 and T2 derived EFC, and electric field magnitude. While head volume was strongly positively associated to T1 and T2 derived EFC, it not directly linked to electric field magnitude. The direct association between age group and electric field magnitude remained substantial even after accounting for both head volume and EFC, indicating that age-related differences in electric field strength cannot be fully explained by differences in head size or image quality alone. Standardized beta coefficients shown with p values: *p < 0.05, **p < 0.01, ***p < 0.001

Given that head volume is associated with both head motion [17] and electric field magnitudes [20], we extended our path models to include head volume as an additional covariate. We focused on EFC in the main analysis because it is strongly associated with head motion—a common issue in older adults that affects image quality and can influence electric field estimates. In this model, head volume had direct effect on T1-derived EFC (β = 0.794, 95% CI [0.722, 0.866], *p* < 0.001) and T2-derived EFC (β = 0.679, 95% CI [0.577, 0.782], *p* < 0.001), suggesting that larger head size is associated with higher levels of image blur or motion-related artifacts. In turn, higher T1 EFC was weakly associated with lower electric field magnitude (β = − 0.163, 95% CI [− 0.441, 0.115], *p* = 0.251) and T2 EFC was not significantly associated (β = 0.099, 95% CI [− 0.117, 0.314], *p* = 0.368), and older adults had higher T1 EFC than younger adults (β = − 0.180, 95% CI [− 0.488, 0.128], *p* = 0.253) and higher T2 EFC (β = − 0.189, 95% CI [− 0.299, − 0.079], *p* = 0.001). However, the indirect effect of head volume on electric field magnitude via EFC was relatively small (head volume direct effect on E-field: β = 0.014, 95% CI [− 0.126, 0.154], *p* = 0.846), indicating that EFC did not mediate the relationship between head volume and electric field magnitude. Importantly, the direct effect of age group on electric field magnitude remained even after accounting for both head volume, and T1- and T2-derived EFC (β = 0.444, 95% CI [0.291, 0.597], *p* < 0.001) (Fig. 3). This indicates that the age-related reduction in electric field magnitude cannot be fully explained by differences in either motion-related image quality or anatomical head volume, reinforcing the robustness of our main finding. Results from models including SNR and CNR are reported in the Supplementary Material (Supplementary Fig. 8).

## Discussion

In this study, we investigated whether standardized MRI quality metrics were associated with the magnitude of electric fields (E-field) induced by tDCS in healthy young and older adults. Our key question was whether lower MRI quality in older adults compromises the reliability of E-field estimates, which is critical for individualized dosing strategies. Our findings demonstrate that while image quality—quantified via EFC, SNR, CNR, FWHM, and INU—differed between age groups, these differences only partially mediated the age-related differences in simulated E-field strength. Crucially, the direct effect of age on electric field magnitude persisted even after accounting for image quality and head volume, suggesting that observed age-related reductions in E-field strength are not biased by lower scan quality but rather reflect genuine anatomical changes that accompany aging.

Older participants showed higher EFC and INU and reductions in SNR, CNR, and FWHM indicating an overall decline in MRI quality compared to younger adults. These changes likely reflect a combination of greater head motion and age-related microstructural alterations, such as reduced gray–white matter differentiation [16, 17, 24]. Our findings align with previous reports of age-related differences in image quality across structural and functional MRI, which have similarly linked aging to increased motion artifacts and diminished tissue contrast [18, 23, 29, 39, 40]. Given their potential to bias derived measures, it is critical to determine whether MRI quality differences influence E-field simulations and whether these simulations remain reliable for individualized dosing in older adults. This question is particularly important because dose–response relationships in brain stimulation are increasingly emphasized in cognitive and clinical neuroscience [5, 41, 42]. If image quality substantially compromises simulation accuracy, individualized simulations of electric fields might misestimate their magnitudes in populations with lower scan quality. Prior studies on electric field intensity and plasticity have rarely accounted for image quality, raising concerns that estimates—especially in older adults—may be biased, with implications for dosing and efficacy [43, 44].

To address this concern, we examined whether variability in MRI quality metrics predicts differences in simulated electric field strength. MRI quality metrics were significantly associated with electric field magnitude across participants, suggesting that lower-quality images may influence simulation outcomes. Specifically, increased motion-related artifacts (higher EFC), reduced signal clarity (lower SNR), and diminished tissue contrast (lower CNR) correlated with lower simulated electric field magnitudes. These associations likely reflect the impact of image degradation on tissue segmentation and mesh construction—particularly in regions with complex structural boundaries such as the scalp, skull, and CSF—which are critical for conductivity modeling in FEM pipelines [13, 45]. Together, these findings highlight the potential for MRI quality differences to systematically bias E-field estimates in aging research.

As such, the aim of this study was to determine whether differences in MRI quality account for age-related reductions in simulated electric field (E-field) magnitudes and whether E-field simulations remain reliable under such conditions. To address this, we tested structural equation models with EFC, SNR, and CNR as mediators of the age–E-field relationship. Adjusting for EFC and SNR had only a minimal effect on the age-group difference in E-field magnitude, indicating that these quality factors do not account for the observed reductions in older adults.

While overall results demonstrate robustness of electric-field estimates across image quality variations, some metric-specific effects warrant consideration. In particular, correction for CNR led to reduced age-group differences in several conditions. This is expected, as CNR quantifies the contrast between gray and white matter, which is known to decline with age due to neurobiological changes such as cortical thinning, increased partial volume effects, and altered tissue properties. Consequently, CNR captures not only technical image quality but also biologically meaningful age-related variance [28, 29]. Thus, lower CNR in older adults may reflect both image quality and anatomical properties relevant to electric field distribution. Consequently, adjusting for CNR may partially remove variance that is intrinsically age-related rather than reflecting image quality.

Effects associated with spatial resolution (FWHM) and intensity non-uniformity (INU) were less pronounced and appeared only in specific conditions. Importantly, these corrections did not systematically eliminate group differences, indicating that electric-field estimates are not strongly driven by resolution or intensity homogeneity within the range observed in this dataset. The consistently smaller age effects observed for P3 simulations across all quality metrics suggest that montage geometry and resulting field distributions play a more substantial role than image quality in determining sensitivity to group differences. This highlights the importance of considering montage-specific factors when interpreting electric-field modeling results [6, 46].

Importantly, these findings indicate that moderate variations in MRI quality do not substantially compromise the reliability of individualized FEM simulations. Even in the presence of elevated EFC and reduced SNR—conditions commonly encountered in aging studies—electric field models retained sensitivity to biologically meaningful group differences. Adding head size as a covariate—given its relevance for both image quality (particularly EFC) and E-field magnitude [17, 18, 23], further confirmed that individualized FEM simulations are reliable to moderate variations in MRI quality. Therefore, while accounting for MRI quality can reduce unexplained variance, our results suggest that it is not strictly necessary for detecting age-related effects in E-field magnitude under typical research conditions. Although we focused on five commonly used metrics (EFC, SNR, CNR, FWHM, and INU), additional MRIQC measures such as CJV, FBER, and WM2MAX were evaluated in pilot analyses. These additional metrics showed very similar associations with electric field magnitude and were highly intercorrelated with our primary metrics (see Supplementary Fig. 9). Thus, our conclusions may not depend on the specific choice of quality metric. This highlights the reliability of well-constructed simulation pipelines and supports their continued use for individualized dosing in aging research and clinical applications, even in the presence of lower scan quality.

## Limitations

This study has several limitations that should be considered when interpreting the findings. First, the age ranges within each group were relatively narrow (YG: 20–35 years; OG: 60–79 years) to ensure homogeneity for group comparisons, which may limit generalizability to the broader aging spectrum, particularly to middle-aged adults (36–59 years) and the oldest-old (80 + years). Second, while the sex distribution was relatively balanced (YG: 59.6% female; OG: 61% female), the modest sample size (n = 106) may limit our ability to detect subtle sex-by-age interaction effects on electric field distributions. Previous research has shown that sex differences in skull thickness and CSF volumes can influence transcranial stimulation field strengths [47], warranting future investigation of sex-age interactions in larger cohorts. Third, participants were cognitively healthy community-dwelling adults, and findings may not generalize to clinical populations or individuals with significant brain pathology. Replication in diverse cohorts, with different scanners, and stimulation parameters, will increase generalizability of our results. SNR and CNR were derived from brain tissue masks, whereas accurate E-field simulations also depend on segmentation of extracerebral tissues such as skull, scalp, and CSF. We assumed that these brain-based quality metrics reflect global scan quality, but this assumption has not been empirically validated. Future studies should include metrics targeting extracerebral structures or direct segmentation accuracy assessments to confirm these findings.

## Conclusions and outlook

Our findings demonstrate that while MRI quality varies significantly with age and influences electric field estimates, it does not explain the reliable age-related reduction in simulated E-field magnitude. Even after adjusting for both image quality and head volume, older adults consistently showed lower E-field strength, suggesting that these differences primarily reflect anatomical and physiological changes rather than imaging artifacts. Critically, this means that E-field simulations remain sufficiently reliable for individualized dose adjustment across the adult lifespan, despite the presence of motion-related artifacts and reduced image quality in older adults. This distinguishes FEM-based modeling from other neuroimaging measures, such as resting-state connectivity, where motion might potentially even invalidate group comparisons.

Future studies could benefit from multimodal MRI approaches to enhance both anatomical and functional precision. While we used T1- and T2-weighted imaging for accurate tissue segmentation, diffusion tensor imaging (DTI) would enable subject-specific anisotropic conductivity modeling in white and gray matter, which can substantially affect electric field distributions [48, 49]. Moreover, functional MRI could provide complementary insights beyond anatomical modeling. Task-based or resting-state fMRI could guide individualized functional localization of stimulation targets and enable investigation of whether electric field strength at functionally active regions relates more strongly to behavioral outcomes than field strength at anatomically defined targets [41]. Integrating functional connectivity measures with field simulations could also help identify whether age-related differences in field distributions preferentially affect functionally connected networks, providing a more mechanistic understanding of individual response variability in brain stimulation interventions.

## Supplementary Information

Below is the link to the electronic supplementary material.

Supplementary Material 1

## Data Availability

The datasets analysed during the current study are not publicly available due to potential identifying information that could compromise participant privacy, but are available from the corresponding author on reasonable request.
