# Supplementary material for: Reliability of electric field simulations in different age groups: impact of data quality metrics
Source: Brain Inform. 2026 Feb 22;13(1):7. doi: 10.1186/s40708-026-00293-2 (PMC12988111; doi:10.1186/s40708-026-00293-2)
Supplement: Supplementary file 1 — Supplementary Material 1 [file 40708_2026_293_MOESM1_ESM.docx]

**Supplementary Material**

Reliability of electric field simulations in different age groups: Impact of data quality metrics

Dayana Hayek^1*^, Axel Thielscher^2,3^, Ulrike Grittner^4^, Agnes Flöel^1,5^, Daria Antonenko^1*^

^1^ Department of Neurology, Universitätsmedizin Greifswald, Greifswald, Germany

^2^ Department of Health Technology, Technical University of Denmark, Kongens Lyngby, Denmark

^3^ Danish Research Centre for Magnetic Resonance, Department of Radiology and Nuclear Medicine, Copenhagen University Hospital Amager and Hvidovre, Hvidovre, Denmark.

^4^ Charité – Universitätsmedizin Berlin, Humboldt-Universität zu Berlin, Berlin Institute of Health, Institute of Biometry and Clinical Epidemiology, 10117 Berlin, Germany

^5^ German Centre for Neurodegenerative Diseases (DZNE) Standort Greifswald, Greifswald, Germany

* *Corresponding authors.* Daria Antonenko, Department of Neurology, University Medicine Greifswald, Ferdinand-Sauerbruch-Straße, 17475 Greifswald, [daria.antonenko@med.uni-greifswald.de](mailto:daria.antonenko@med.uni-greifswald.de). Dayana Hayek, Department of Neurology, University Medicine Greifswald, Ferdinand-Sauerbruch-Straße, 17475 Greifswald, [dayana.hayek@med.uni-greifswald.de](mailto:dayana.hayek@med.uni-greifswald.de).

**
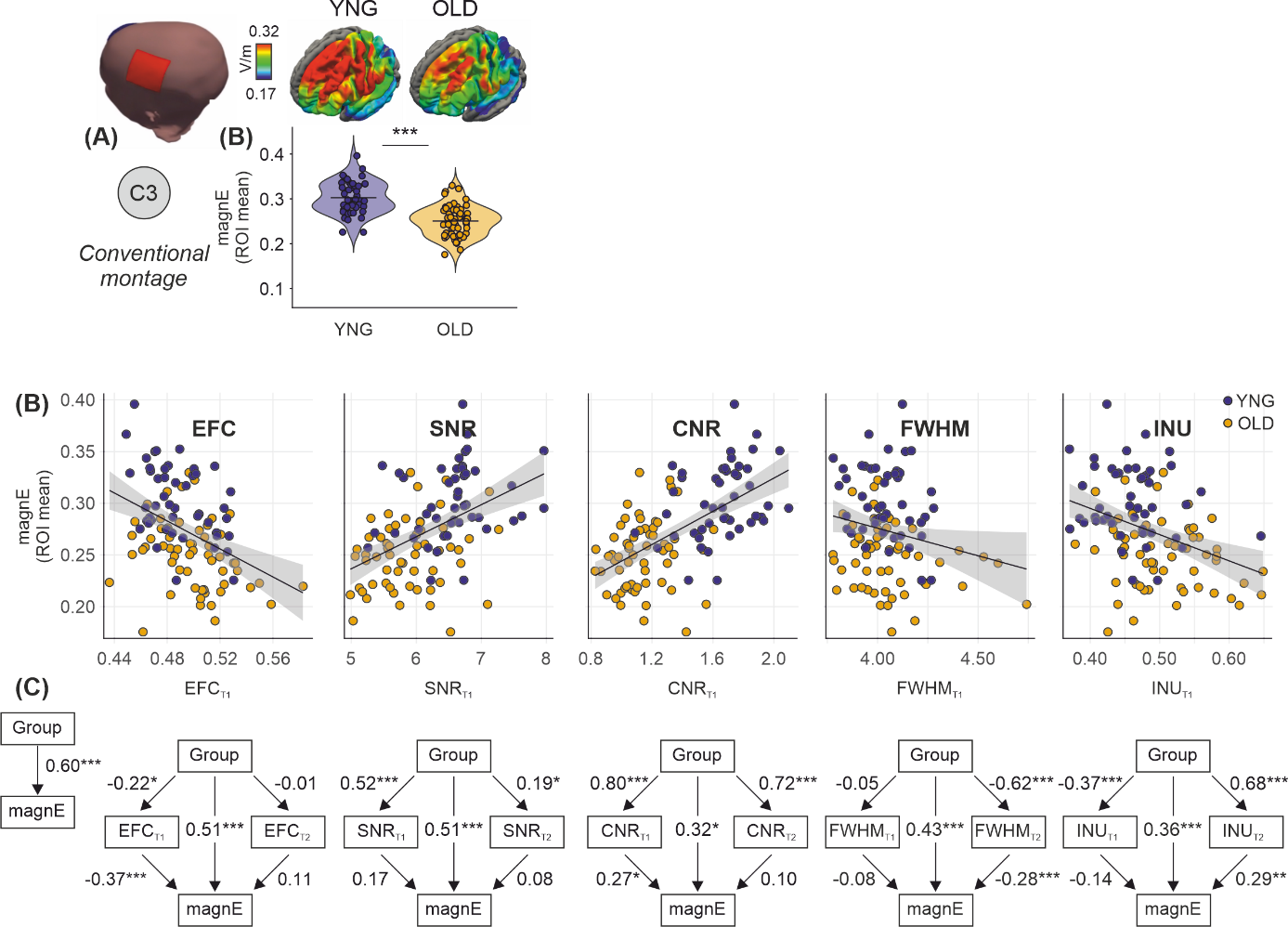
**

**Supplementary Figure 1. Age-Related Differences in Electric Field Magnitude and Mediation by MRI Quality Metrics (Conventional C3 Montage). (A) Electrode placement for the conventional C3 montage (left), group-averaged electric field magnitude distributions for younger and older adults (middle), and violin plots comparing electric field magnitude between groups (right), showing significantly higher values in younger adults (***p < 0.001). (B) Scatter plots showing associations between electric field magnitude and T1-weighted image quality metrics—Entropy Focus Criterion (EFC), Signal-to-Noise Ratio (SNR), Contrast-to-Noise Ratio (CNR), spatial resolution (full-width-half-maximum, FWHM), and intensity uniformity (INU)—across both age groups. (C) Structural Equation Models (SEMs) illustrating the direct and indirect effects of age group on electric field magnitude via MRI quality metrics from both T1- and T2-weighted images (EFC, SNR, CNR, FWHM, and INU). For this montage, age group significantly predicted electric field magnitude. The direct effect remained significant after adjustment for EFC, SNR, CNR, FWHM, and INU. Standardized path coefficients are shown with p-values: *p < 0.05, **p < 0.01, ***p < 0.001.**


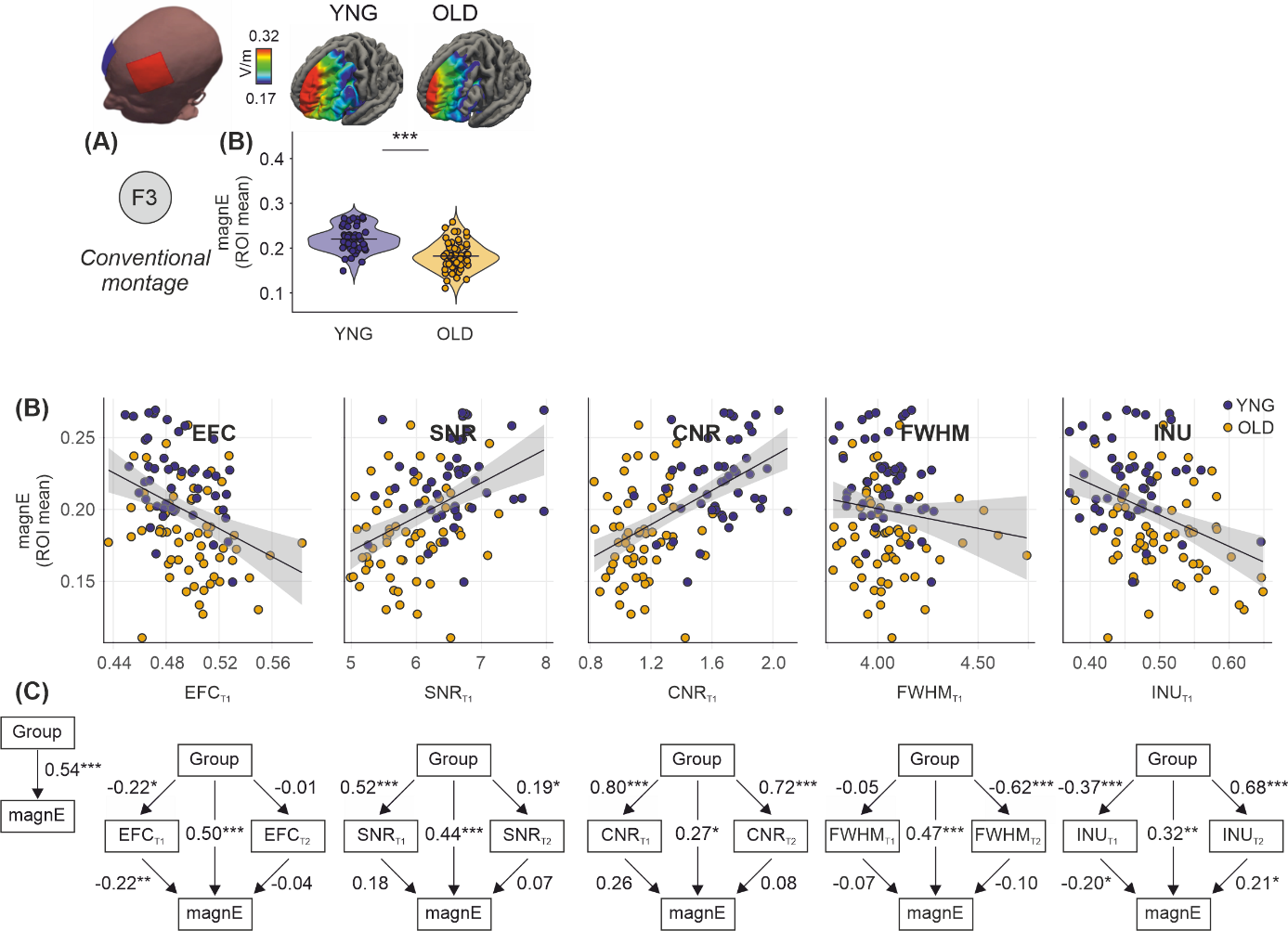


**Supplementary Figure 2. Age-Related Differences in Electric Field Magnitude and Mediation by MRI Quality Metrics (Conventional F3 Montage). (A)** Electrode placement for the conventional F3 montage (left), group-averaged electric field magnitude distributions for younger and older adults (middle), and violin plots comparing electric field magnitude between groups (right), showing significantly higher values in younger adults (***p*** < 0.001). **(B)** Scatter plots showing associations between electric field magnitude and T1-weighted image quality metrics—Entropy Focus Criterion (EFC), Signal-to-Noise Ratio (SNR), and Contrast-to-Noise Ratio (CNR), **spatial resolution (full-width-half-maximum, FWHM), and intensity uniformity (INU)—across both age groups. (C) Structural Equation Models (SEMs) illustrating the direct and indirect effects of age group on electric field magnitude via MRI quality metrics from both T1- and T2-weighted images (EFC, SNR, CNR, FWHM, and INU). For this montage, age group significantly predicted electric field magnitude. The direct effect remained significant after adjustment for EFC, SNR, CNR, FWHM, and INU.** For this montage, age group significantly predicted electric field magnitude. The direct effect remained significant after adjustment for EFC, SNR but not for CNR. Standardized path coefficients are shown with p-values: *p < 0.05, **p < 0.01, ***p < 0.001.


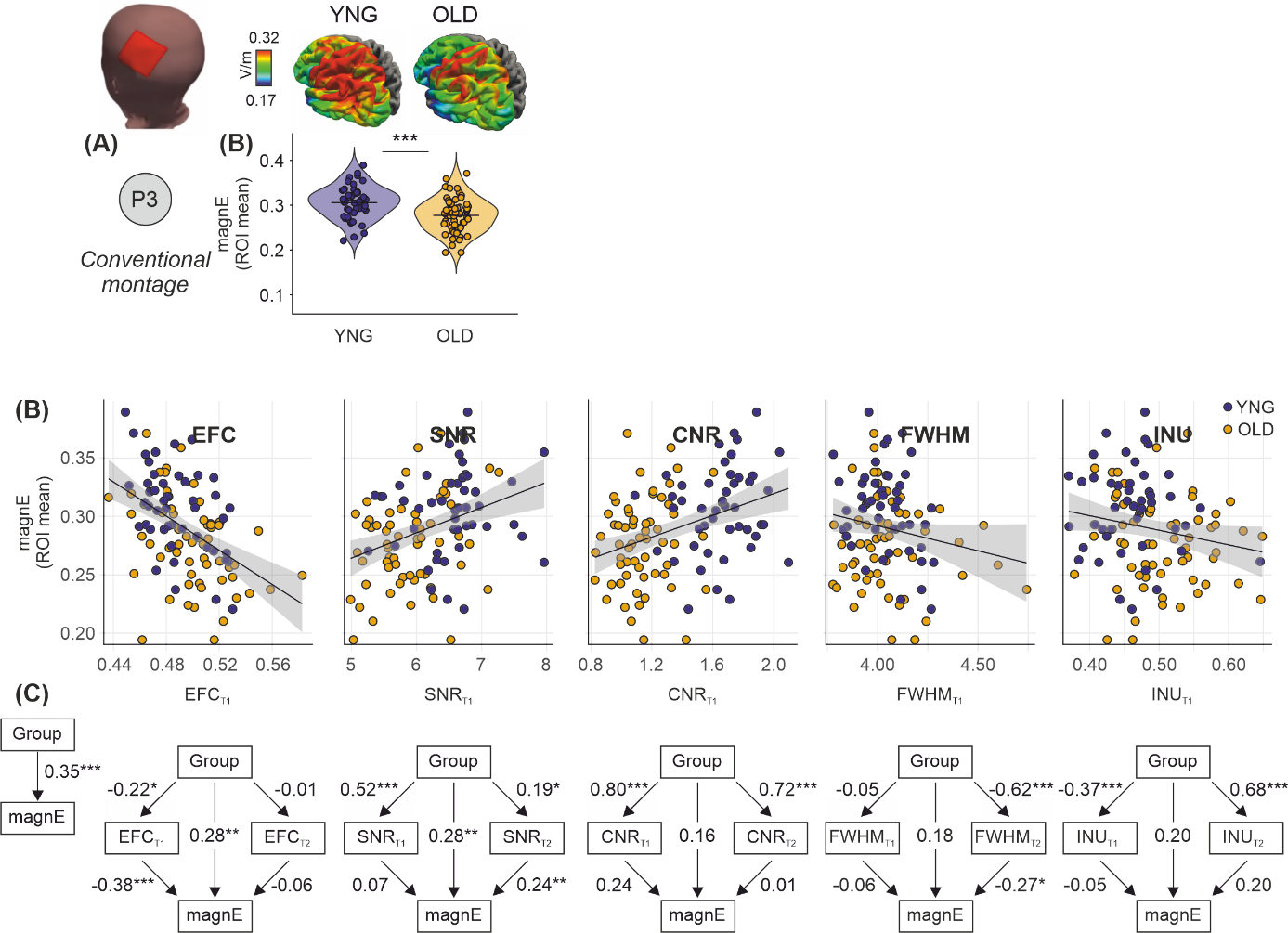


**Supplementary Figure 3.** Age-Related Differences in Electric Field Magnitude and Mediation by MRI Quality Metrics (Conventional P3 Montage). **(A)** Electrode placement for the conventional P3 montage (left), group-averaged electric field magnitude distributions for younger and older adults (middle), and violin plots comparing electric field magnitude between groups (right), showing significantly higher values in younger adults (*p* < 0.001). **(B)** Scatter plots showing associations between electric field magnitude and T1-weighted image quality metrics—Entropy Focus Criterion (EFC), Signal-to-Noise Ratio (SNR), and Contrast-to-Noise Ratio (CNR), **spatial resolution (full-width-half-maximum, FWHM), and intensity uniformity (INU)—across both age groups. (C) Structural Equation Models (SEMs) illustrating the direct and indirect effects of age group on electric field magnitude via MRI quality metrics from both T1- and T2-weighted images (EFC, SNR, CNR, FWHM, and INU). For this montage, age group significantly predicted electric field magnitude. The direct effect remained significant after adjustment for EFC, SNR, CNR, FWHM, and INU.** For this montage, age group significantly predicted electric field magnitude. The direct effect remained significant after adjustment for EFC, SNR but not for CNR. Standardized path coefficients are shown with p-values: *p < 0.05, **p < 0.01, ***p < 0.001.


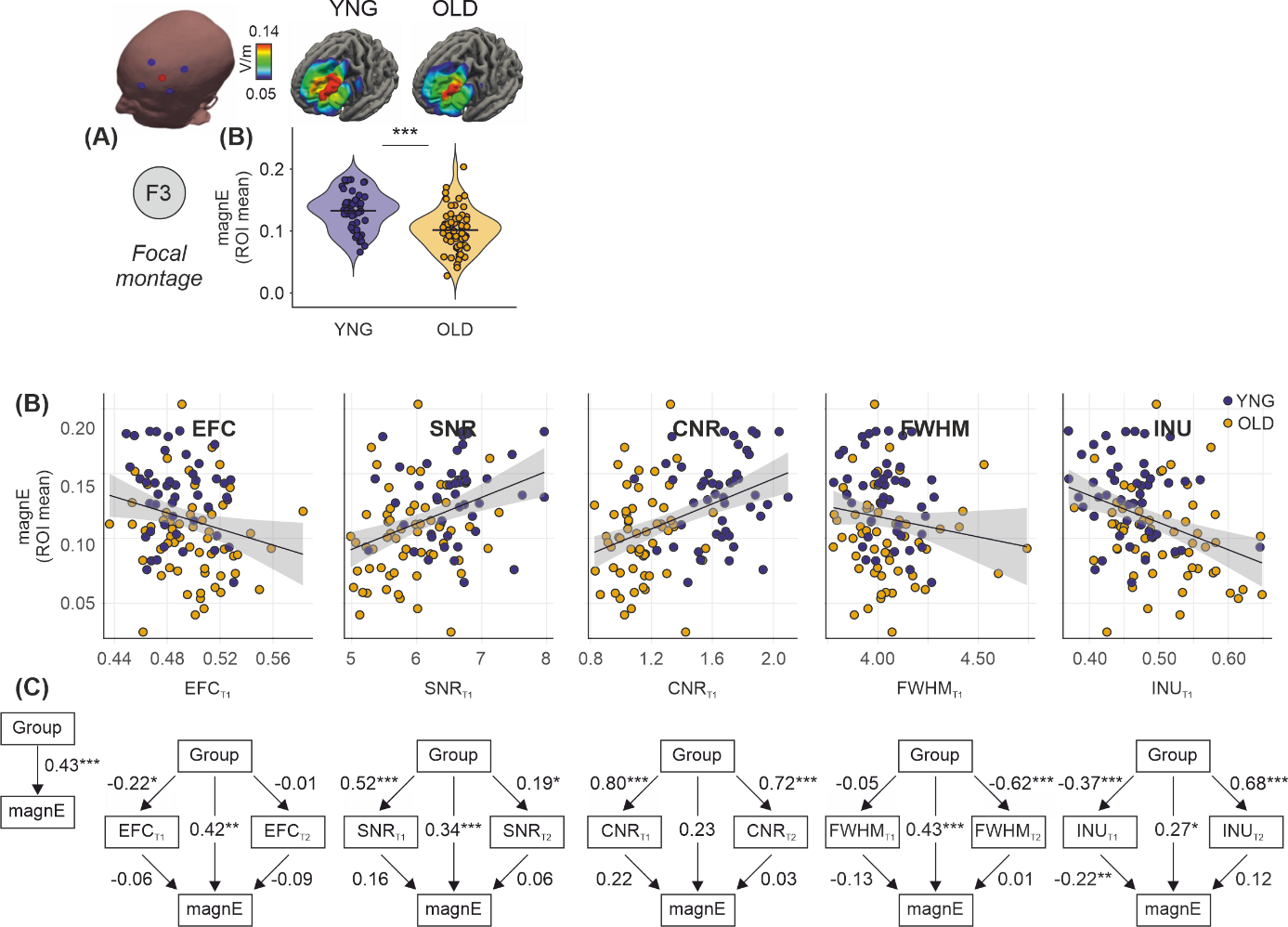


**Supplementary Figure 4.** Age-Related Differences in Electric Field Magnitude and Mediation by MRI Quality Metrics (Focal F3 Montage). **(A)** Electrode placement for the focal F3 montage (left), group-averaged electric field magnitude distributions for younger and older adults (middle), and violin plots comparing electric field magnitude between groups (right), showing significantly higher values in younger adults (*p* < 0.001). **(B)** Scatter plots showing associations between electric field magnitude and T1-weighted image quality metrics—Entropy Focus Criterion (EFC), Signal-to-Noise Ratio (SNR), and Contrast-to-Noise Ratio (CNR), **spatial resolution (full-width-half-maximum, FWHM), and intensity uniformity (INU)—across both age groups. (C) Structural Equation Models (SEMs) illustrating the direct and indirect effects of age group on electric field magnitude via MRI quality metrics from both T1- and T2-weighted images (EFC, SNR, CNR, FWHM, and INU). For this montage, age group significantly predicted electric field magnitude. The direct effect remained significant after adjustment for EFC, SNR, CNR, FWHM, and INU.** For this montage, age group significantly predicted electric field magnitude. The direct effect remained significant after adjustment for EFC, SNR but not for CNR. Standardized path coefficients are shown with p-values: *p < 0.05, **p < 0.01, ***p < 0.001.


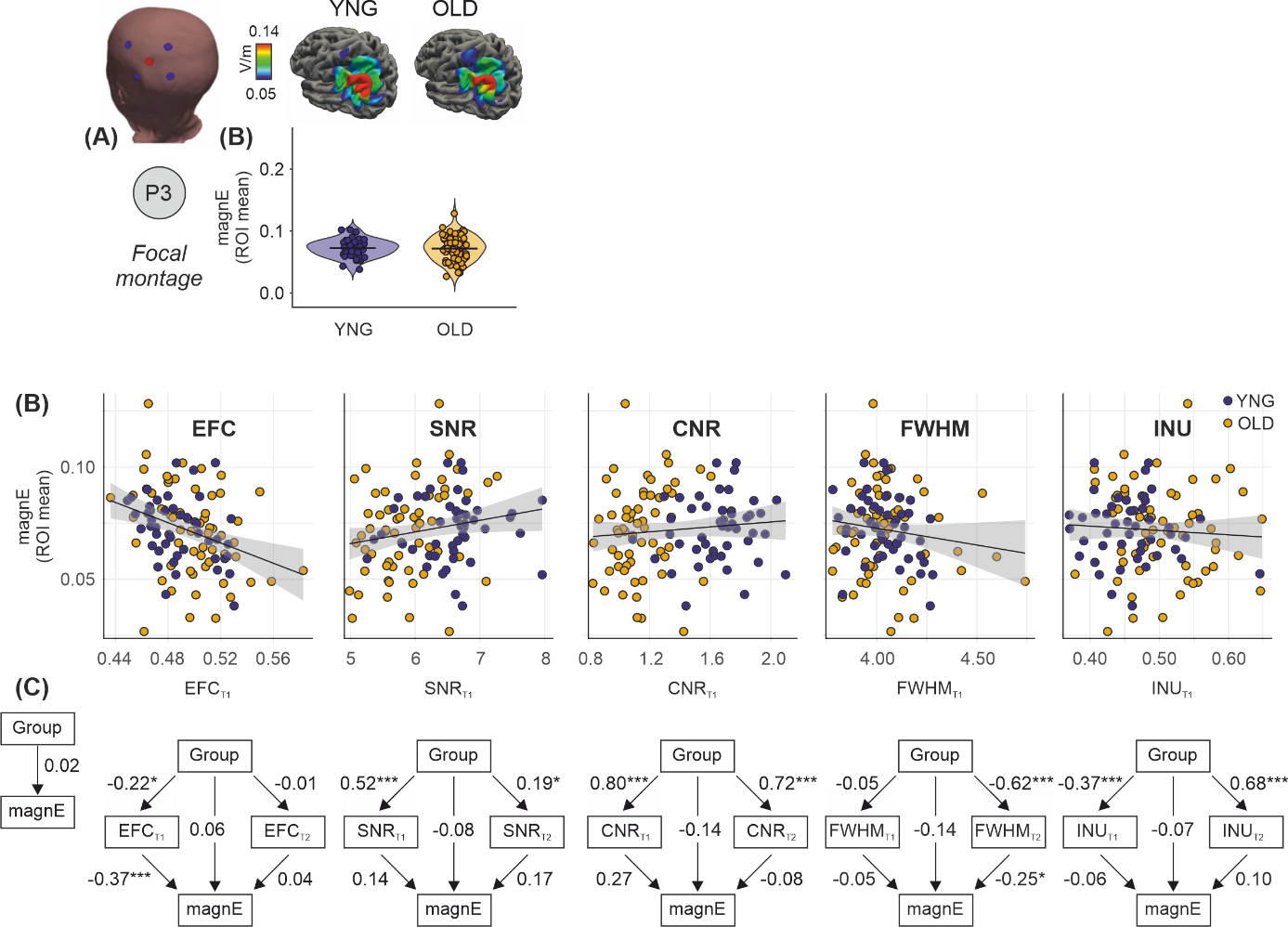


**Supplementary Figure 5.** Age-Related Differences in Electric Field Magnitude and Mediation by MRI Quality Metrics (Focal P3 Montage). **(A)** Electrode placement for the focal P3 montage (left), group-averaged electric field magnitude distributions for younger and older adults (middle), and violin plots comparing electric field magnitude between groups (right), showing no difference between young and old. **(B)** Scatter plots showing associations between electric field magnitude and T1-weighted image quality metrics—Entropy Focus Criterion (EFC), Signal-to-Noise Ratio (SNR), and Contrast-to-Noise Ratio (CNR), **spatial resolution (full-width-half-maximum, FWHM), and intensity uniformity (INU)—across both age groups. (C) Structural Equation Models (SEMs) illustrating the direct and indirect effects of age group on electric field magnitude via MRI quality metrics from both T1- and T2-weighted images (EFC, SNR, CNR, FWHM, and INU). For this montage, age group significantly predicted electric field magnitude. The direct effect remained significant after adjustment for EFC, SNR, CNR, FWHM, and INU.** For this montage, age group did not predict electric field magnitude. The direct effect was not significant after adjustment for EFC, SNR, and CNR. Standardized path coefficients are shown with p-values: *p < 0.05, **p < 0.01, ***p < 0.001.

**
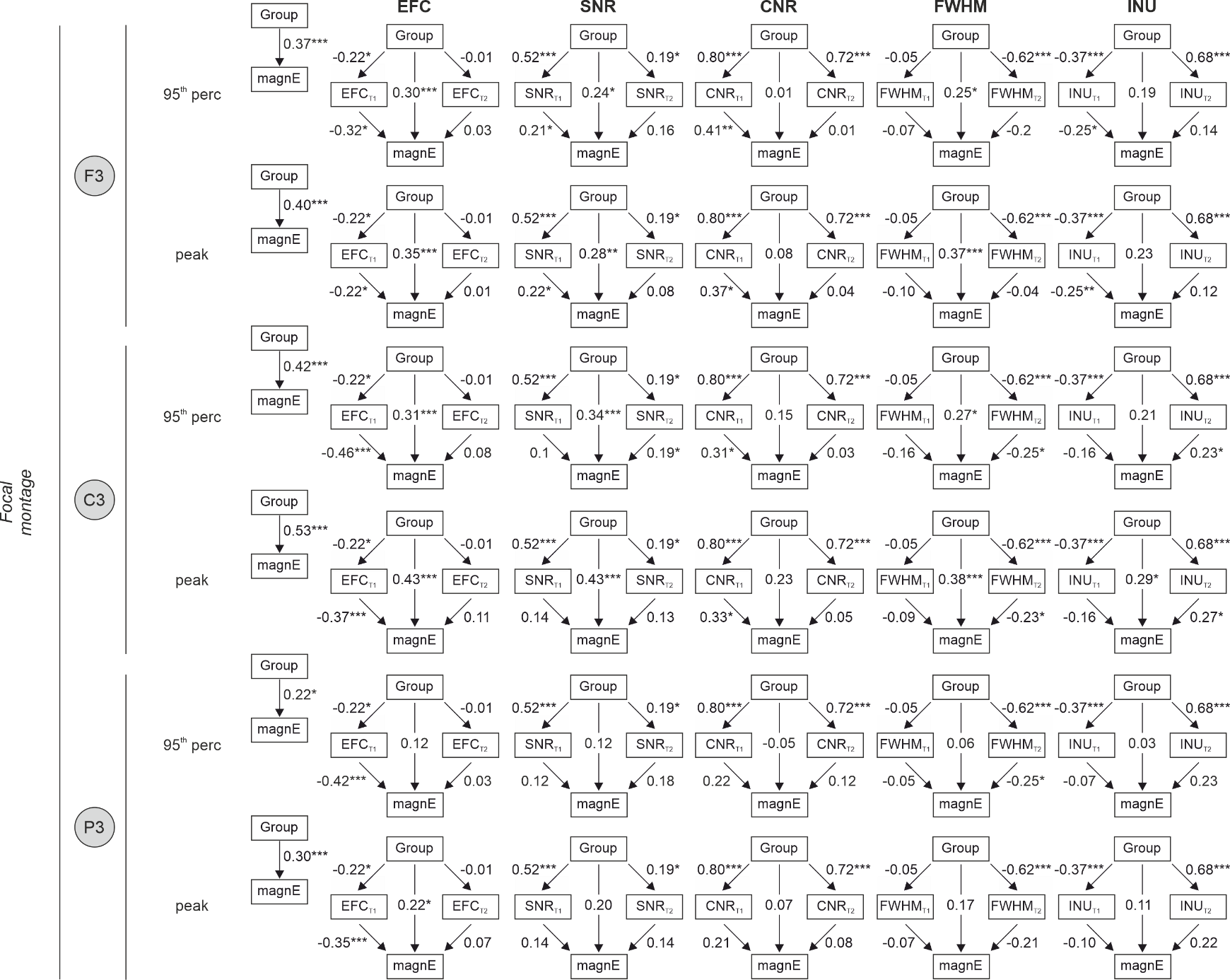
Supplementary Figure 6. Structural Equation Models (SEMs) illustrating the direct and indirect effects of age group on electric field magnitude for focal montages (95^th^ percentiles and peak values) via MRI quality metrics from both T1- and T2-weighted images (EFC, SNR, CNR, FWHM, and INU). EFC, entropy focus criterion. SNR, signal to noise ratio. CNR, contrast to noise ratio. FWHM, full width at half maximum. INU, intensity uniformity. Standardized path coefficients are shown with p-values: *p < 0.05, **p < 0.01, ***p < 0.001.**

**
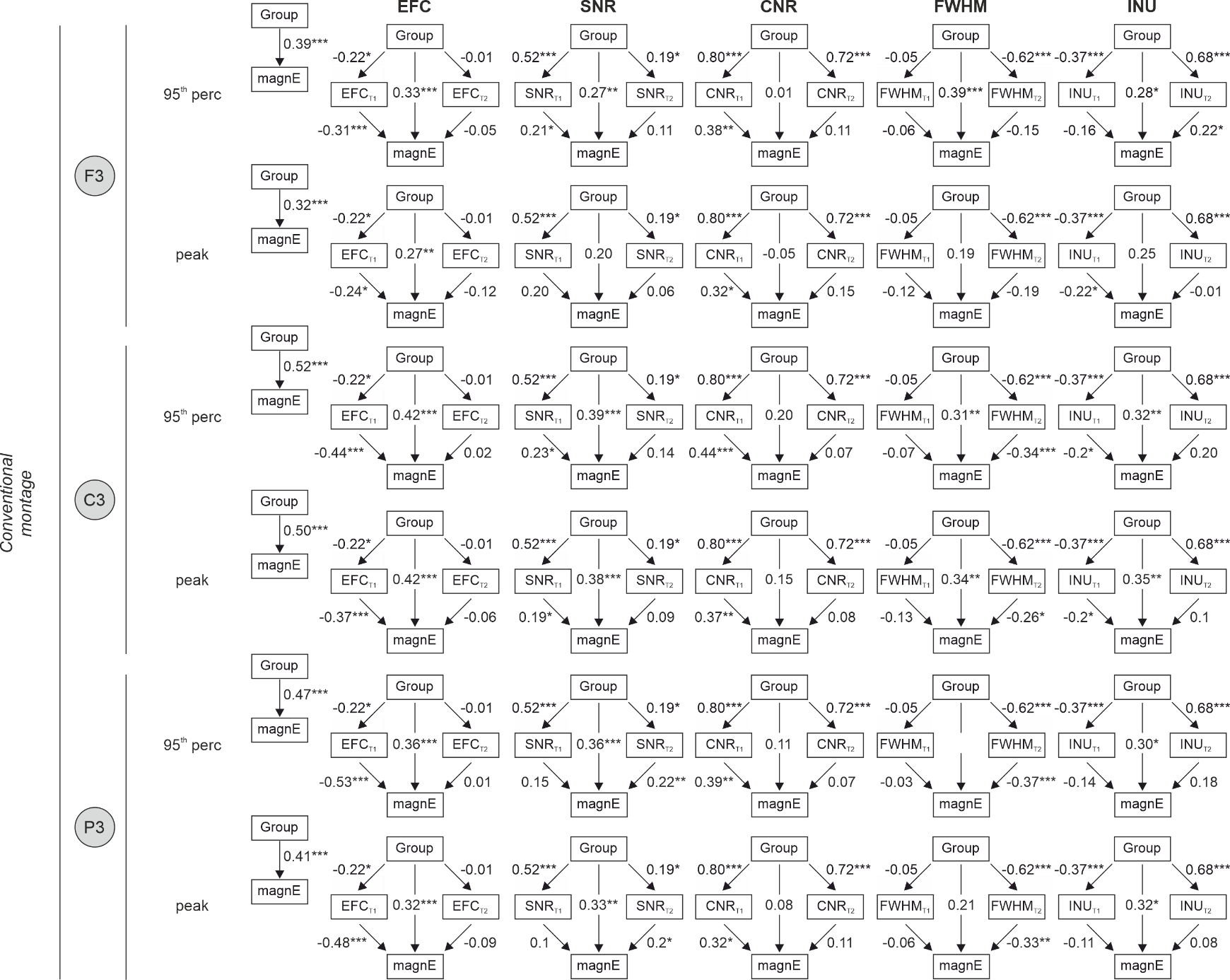
Supplementary Figure 7. Structural Equation Models (SEMs) illustrating the direct and indirect effects of age group on electric field magnitude for conventional montages (95^th^ percentiles and peak values) via MRI quality metrics from both T1- and T2-weighted images (EFC, SNR, CNR, FWHM, and INU). EFC, entropy focus criterion. SNR, signal to noise ratio. CNR, contrast to noise ratio. FWHM, full width at half maximum. INU, intensity uniformity. Standardized path coefficients are shown with p-values: *p < 0.05, **p < 0.01, ***p < 0.001.**


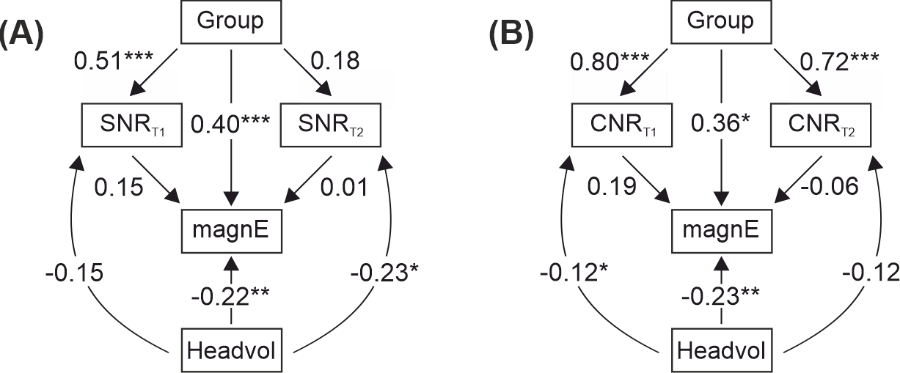


**Supplementary Figure 8**. Structural Equation Models Evaluating Head Volume and Image Quality Metrics as Mediators of Electric Field Magnitude. **(A)** SEM illustrating the relationships among age group, head volume, and MRI quality metrics (T1- and T2-derived Signal-to-Noise Ratio, SNR) in predicting electric field magnitude. While head volume was negatively associated with electric field magnitude (*β* = –0.217, p < 0.01), it was not a strong mediator, and its indirect pathways via SNR were small. Age group remained significantly associated with electric field magnitude (*β* = 0.399, p < 0.001) even after accounting for both head volume and image quality, indicating that age-related differences cannot be fully explained by head size or scan quality alone. **(B)** SEM evaluating the same relationships but with Contrast-to-Noise Ratio (CNR) from T1- and T2-weighted images as mediators. Head volume showed a small negative association with electric field magnitude (*β* = –0.227, p < 0.01), while the paths through CNR were weak. Age group continued to have a substantial direct effect on electric field magnitude (*β* = 0.364, *p* < 0.05), again suggesting that anatomical and biological factors beyond image quality and head size drive these differences. Standardized beta coefficients are shown with p-values: *p < 0.05, **p < 0.01, ***p < 0.001.


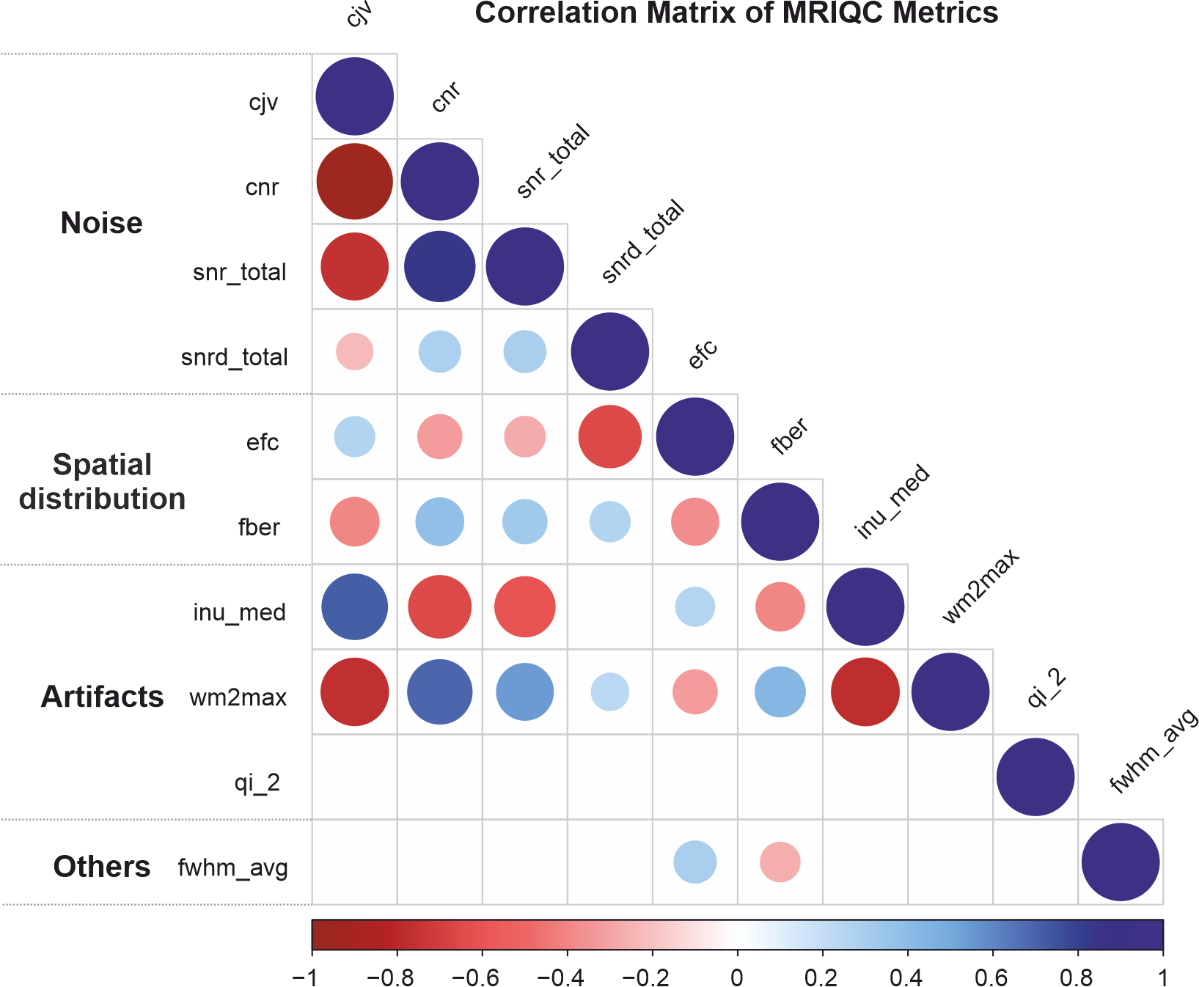


**Supplementary Figure 7**. Correlation Matrix of MRI Quality Metrics Extracted from T1-Weighted Images Using MRIQC. This figure displays pairwise correlations among multiple image quality metrics derived from T1-weighted structural scans using MRIQC. Metrics are grouped into categories: Noise (CJV = Coefficient of Joint Variation, CNR = Contrast-to-Noise Ratio, SNR = Signal-to-Noise Ratio), Spatial Distribution (EFC = Entropy Focus Criterion, FBER = Foreground-Background Energy Ratio), Artifacts (INU = Intensity Non-Uniformity, WM2MAX = White Matter to Maximum Intensity Ratio, QI2 = Second-order Quality Index), and Others (FWHM = Full Width at Half Maximum). The size and color intensity of each circle indicate the strength and direction of Pearson correlations (range: –1 to 1), with blue representing positive and red representing negative correlations. Strong positive correlations are evident among noise-related metrics (CJV, CNR, SNR), while EFC and FBER show moderate associations with noise measures. This matrix highlights the interdependencies among quality metrics, emphasizing the importance of accounting for multicollinearity when incorporating these measures into statistical models.

**Robustness of electric-field estimates across image quality**

To directly assess robustness to image quality, we conducted median-split stratified analyses based on T1 quality metrics (EFC, SNR, CNR, FWHM, INU). For each target (C3, F3, P3) and montage (focal, conventional), age-group differences in ROI-mean electric-field magnitude were estimated separately in low- and high-quality strata. For C3 and F3 simulations, age effects were highly consistent across quality strata. In focal montages, group differences remained significant in both low- and high-quality halves for all quality metrics (β ≈ 0.02–0.04), with comparable effect sizes across strata (Cohen’s d ≈ 0.8–1.3). Conventional montages showed even larger and highly stable effects (β ≈ 0.04–0.06; Cohen’s d often > 1.3), again with no evidence of attenuation in lower-quality data. Formal interaction tests revealed no significant Group × Quality interactions for any metric, montage, or target (Supplementary Table 1), indicating that age effects were not systematically modulated by image quality. In contrast, P3 simulations showed smaller or absent age effects. These patterns were consistent across low- and high-quality strata, suggesting that the reduced sensitivity in P3 reflects simulation-specific factors rather than image quality. Together, these results demonstrate that group-level electric-field estimates are robust across a wide range of image quality, providing direct evidence for sufficient reliability even under lower-quality imaging conditions.

**Supplementary Table 1.** Median-split stratified analyses of age effects on electric field magnitude (ROI mean) across T1 image quality metrics. β coefficients represent Young–Old differences estimated separately within low- and high-quality strata. No significant Group × Quality interactions were observed, indicating stable age effects across image quality.

| **Focal** |  | **Quality** | **Stratum** | **β** | **95% CI** | **p** | **Cohen’s d** | **p(Group×Quality)** |
| --- | --- | --- | --- | --- | --- | --- | --- | --- |
|  | C3 | EFC | Low | 0.029 | [0.014, 0.044] | <.001 | 1.08 | 0.60 |
|  |  |  | High | 0.024 | [0.010, 0.038] | .001 | 0.97 |  |
|  |  | SNR | Low | 0.024 | [0.009, 0.040] | .002 | 1.13 | 0.97 |
|  |  |  | High | 0.025 | [0.007, 0.043] | .008 | 0.82 |  |
|  |  | CNR | High | 0.030 | [0.009, 0.052] | .007 | 1.03 | 0.54 |
|  |  | FWHM | Low | 0.025 | [0.012, 0.038] | <.001 | 1.07 | 0.33 |
|  |  |  | High | 0.035 | [0.019, 0.051] | <.001 | 1.22 |  |
|  |  | INU | Low | 0.026 | [0.010, 0.042] | .003 | 0.88 | 0.70 |
|  |  |  | High | 0.030 | [0.016, 0.044] | <.001 | 1.30 |  |
|  | F3 | EFC | Low | 0.027 | [0.009, 0.045] | .005 | 0.81 | 0.76 |
|  |  |  | High | 0.031 | [0.012, 0.050] | .002 | 0.97 |  |
|  |  | SNR | Low | 0.028 | [0.004, 0.053] | .024 | 0.82 | 0.97 |
|  |  |  | High | 0.028 | [0.009, 0.046] | .005 | 0.89 |  |
|  |  | CNR | High | 0.019 | [−0.006, 0.045] | .138 | 0.55 | 0.57 |
|  |  | FWHM | Low | 0.031 | [0.012, 0.050] | .002 | 0.93 | 0.87 |
|  |  |  | High | 0.033 | [0.016, 0.051] | <.001 | 1.05 |  |
|  |  | INU | Low | 0.028 | [0.011, 0.045] | .001 | 0.94 | 0.90 |
|  |  |  | High | 0.030 | [0.009, 0.051] | .007 | 0.84 |  |
|  | P3 | EFC | Low | −0.006 | [−0.016, 0.004] | .204 | −0.36 | 0.15 |
|  |  |  | High | 0.004 | [−0.006, 0.013] | .441 | 0.23 |  |
|  |  | SNR | Low | 0.004 | [−0.007, 0.016] | .461 | 0.26 | 0.09 |
|  |  |  | High | −0.009 | [−0.020, 0.002] | .096 | −0.51 |  |
|  |  | CNR | High | −0.005 | [−0.017, 0.007] | .432 | −0.29 | 0.67 |
|  |  | FWHM | Low | 0.002 | [−0.008, 0.012] | .735 | 0.10 | 0.87 |
|  |  |  | High | 0.001 | [−0.010, 0.011] | .919 | 0.03 |  |
|  |  | INU | Low | −0.000 | [−0.011, 0.010] | .934 | −0.02 | 0.68 |
|  |  |  | High | 0.003 | [−0.008, 0.013] | .632 | 0.14 |  |
| Conv | C3 | EFC | Low | 0.049 | [0.030, 0.068] | <.001 | 1.44 | 0.83 |
|  |  |  | High | 0.046 | [0.027, 0.066] | <.001 | 1.42 |  |
|  |  | SNR | Low | 0.039 | [0.017, 0.061] | <.001 | 1.25 | 0.45 |
|  |  |  | High | 0.051 | [0.029, 0.074] | <.001 | 1.37 |  |
|  |  | CNR | High | 0.053 | [0.025, 0.081] | <.001 | 1.38 | 0.22 |
|  |  | FWHM | Low | 0.048 | [0.031, 0.065] | <.001 | 1.59 | 0.45 |
|  |  |  | High | 0.058 | [0.038, 0.079] | <.001 | 1.55 |  |
|  |  | INU | Low | 0.053 | [0.032, 0.073] | <.001 | 1.46 | 0.80 |
|  |  |  | High | 0.049 | [0.029, 0.069] | <.001 | 1.46 |  |
|  | F3 | EFC | Low | 0.036 | [0.020, 0.052] | <.001 | 1.25 | 0.86 |
|  |  |  | High | 0.034 | [0.016, 0.051] | <.001 | 1.13 |  |
|  |  | SNR | Low | 0.032 | [0.010, 0.053] | .004 | 1.05 | 0.97 |
|  |  |  | High | 0.031 | [0.014, 0.049] | <.001 | 1.07 |  |
|  |  | CNR | High | 0.034 | [0.012, 0.056] | .003 | 1.15 | 0.44 |
|  |  | FWHM | Low | 0.037 | [0.020, 0.055] | <.001 | 1.19 | 0.84 |
|  |  |  | High | 0.040 | [0.024, 0.055] | <.001 | 1.41 |  |
|  |  | INU | Low | 0.033 | [0.017, 0.049] | <.001 | 1.14 | 0.52 |
|  |  |  | High | 0.041 | [0.022, 0.059] | <.001 | 1.30 |  |
|  | P3 | EFC | Low | 0.016 | [−0.005, 0.037] | .124 | 0.43 | 0.35 |
|  |  |  | High | 0.029 | [0.010, 0.049] | .003 | 0.89 |  |
|  |  | SNR | Low | 0.028 | [0.005, 0.051] | .017 | 0.86 | 0.54 |
|  |  |  | High | 0.017 | [−0.008, 0.043] | .183 | 0.40 |  |
|  |  | CNR | High | 0.020 | [−0.010, 0.050] | .185 | 0.49 | 0.66 |
|  |  | FWHM | Low | 0.029 | [0.009, 0.049] | .005 | 0.82 | 0.93 |
|  |  |  | High | 0.030 | [0.009, 0.052] | .007 | 0.77 |  |
|  |  | INU | Low | 0.025 | [0.002, 0.047] | .031 | 0.62 | 0.54 |
|  |  |  | High | 0.034 | [0.012, 0.056] | .003 | 0.93 |  |
